# Pylorus preserving loop duodeno-enterostomy with sleeve gastrectomy - preliminary results

**DOI:** 10.1186/1471-2482-14-20

**Published:** 2014-04-11

**Authors:** Jodok Matthias Grueneberger, Iwona Karcz-Socha, Goran Marjanovic, Simon Kuesters, Krystyna Zwirska-Korczala, Katharina Schmidt, W Konrad Karcz

**Affiliations:** 1 Department of General and Visceral Surgery, University of Freiburg, Hugstetter Strasse 55, 79106 Freiburg, Germany; 2 Department of Physiology, Silesian Medical University, Katowitz, Poland; 3 Department of General Surgery, University of Schleswig-Holstein, Campus Lübeck, Lübeck, Germany

## Abstract

**Background:**

Bariatric operations mostly combine a restrictive gastric component with a rerouting of the intestinal passage. The pylorus can thereby be alternatively preserved or excluded. With the aim of performing a “pylorus-preserving gastric bypass”, we present early results of a proximal postpyloric loop duodeno-jejunostomy associated with a sleeve gastrectomy (LSG) compared to results of a parallel, but distal LSG with a loop duodeno-ileostomy as a two-step procedure.

**Methods:**

16 patients underwent either a two-step LSG with a distal loop duodeno-*ileostomy* (DIOS) as revisional bariatric surgery or a combined single step operation with a proximal duodeno-*jejunostomy* (DJOS). Total small intestinal length was determined to account for inter-individual differences.

**Results:**

Mean operative time for the second-step of the DIOS operation was 121 min and 147 min for the combined DJOS operation. The overall intestinal length was 750.8 cm (range 600-900 cm) with a bypassed limb length of 235.7 cm in DJOS patients. The mean length of the common channel in DIOS patients measured 245.6 cm. Overall excess weight loss (%EWL) of the two-step DIOS procedure came to 38.31% and 49.60%, DJOS patients experienced an %EWL of 19.75% and 46.53% at 1 and 6 months, resp. No complication related to the duodeno-enterostomy occurred.

**Conclusions:**

Loop duodeno-enterosomies with sleeve gastrectomy can be safely performed and may open new alternatives in bariatric surgery with the possibility for inter-individual adaptation.

## Background

Bariatric surgery has proven to be the most effective treatment for long-term weight loss and metabolic rebalancing in obese patients [1,2]. Most procedures combine a restrictive gastric component with a rerouting of the intestinal passage. Prominent examples are the Roux-en-Y gastric bypass (RYGB) or the biliopancreatic diversion (BPD). Gastric restriction either involves the entire stomach therefore preserving the pylorus when reconstructing the intestinal passage, or only the proximal part of the stomach is used to form a gastric pouch thus leaving a remnant stomach. Passage reconstruction then requires a gastro-enterostomy.

Preserving the pylorus when bypassing the duodenum has led to important technical changes in bariatric surgery. In order to avoid a dumping syndrome and marginal ulcers that occasionally occurred after Scopinaro´s initial BPD, Marceau et al. successfully changed the technique to perform a biliopancreatic diversion with duodenal switch (BPD/DS) with similar limb variations, using however a postpyloric reconstruction [3].

The RYGB generally is one of the best established procedures in bariatric surgery [4]. However the failure rate with weight regain due to a dilatation of the gastric pouch, gastro-jejunostomy and proximal jejunum is up to 35% [5]. Recently, bile reflux was identified as one important cause of postoperative pain [6]. Again, a postpyloric reconstruction seems tempting for this procedure.

We here present perioperative data of a proximal (similar to RYGB) and distal (similar to BPD/DS) postpyloric loop duodeno-enterostomy with sleeve gastrectomy. The distal duodeno-enterostomy, based on the earlier described single anastomosis duodeno-ileostomy associated to a sleeve gastectomy (SADI-S) operation [7], was performed as revisionary bariatric operation.

## Methods

### Patients

From October 2011 to September 2012, 16 patients underwent loop duodeno-enterostomies for bariatric surgery. Explicit written informed consent for operation and data recording was obtained from all patients. Data recording and evaluation was approved by the ethics committee of the University of Freiburg (ref. number 321/13) and was in accordance with the Declaration of Helsinki. A proximal duodeno-jejunostomy with sleeve gastrectomy (DJOS) was conducted as an alternative to RYGB in 7 selected patients eligible for bariatric surgery with a body mass index (BMI) range from 35.7 to 47.9 kg/m^2^ (median BMI 42.7 kg/m^2^). In case of previous gastric banding and relevant perigastric scar tissue, instead of a sleeve gastrectomy, a gastric plicature was performed (n = 3/7) to minimize operative risk. Two-step DIOS was performed as revisionary surgery after failed RYGB due to dumping syndrome (n = 2/9) or after sleeve gastrectomy with insufficient weight loss alone (3/9) or in combination with persisting type 2 diabetes (T2DM, 4/9). All operations were performed by the same senior surgeon. In order to prevent vitamin deficiencies, besides a multivitamin, patients are prescribed Calcium (500 mg twice daily), Vitamin D3 (1000 IU daily), folic acid (5 mg daily) and iron (100 mg daily) supplementation.

Data recording included length of hospital stay, preoperative BMI, presence of medical comorbidities, intra- and postoperative complications, management of complications, total operative time, common channel length and weight loss. Total intestinal length was recorded only after February 2012. All data were entered prospectively into a custom-designed database. The patients had the same follow-up protocol at the outpatient clinic at 1, 3, 6, and 12 months after surgery, followed by an annual visit.

### Operative technique

The patient is placed in the split-leg position with the operating surgeon standing between the legs. Trocar positions are similar to those used for banded sleeve gastrectomy [8].

**Figure 1:**
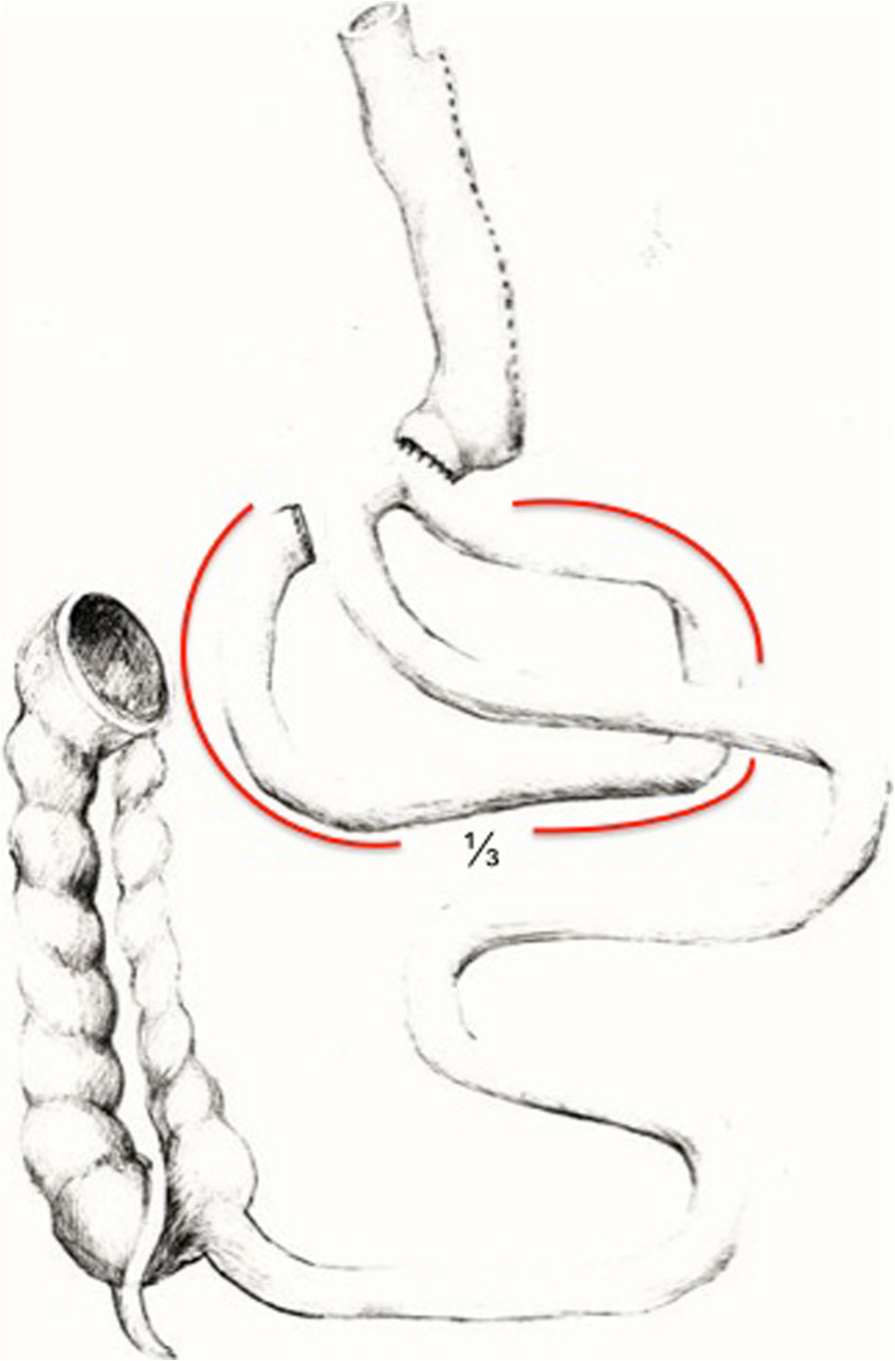
**Diagram of a duodeno-jejunostomy with sleeve gastrectomy (DJOS).** The bypassed intestinal length (1/3 of overall intestinal length) is labelled in red.

**Figure 2:**
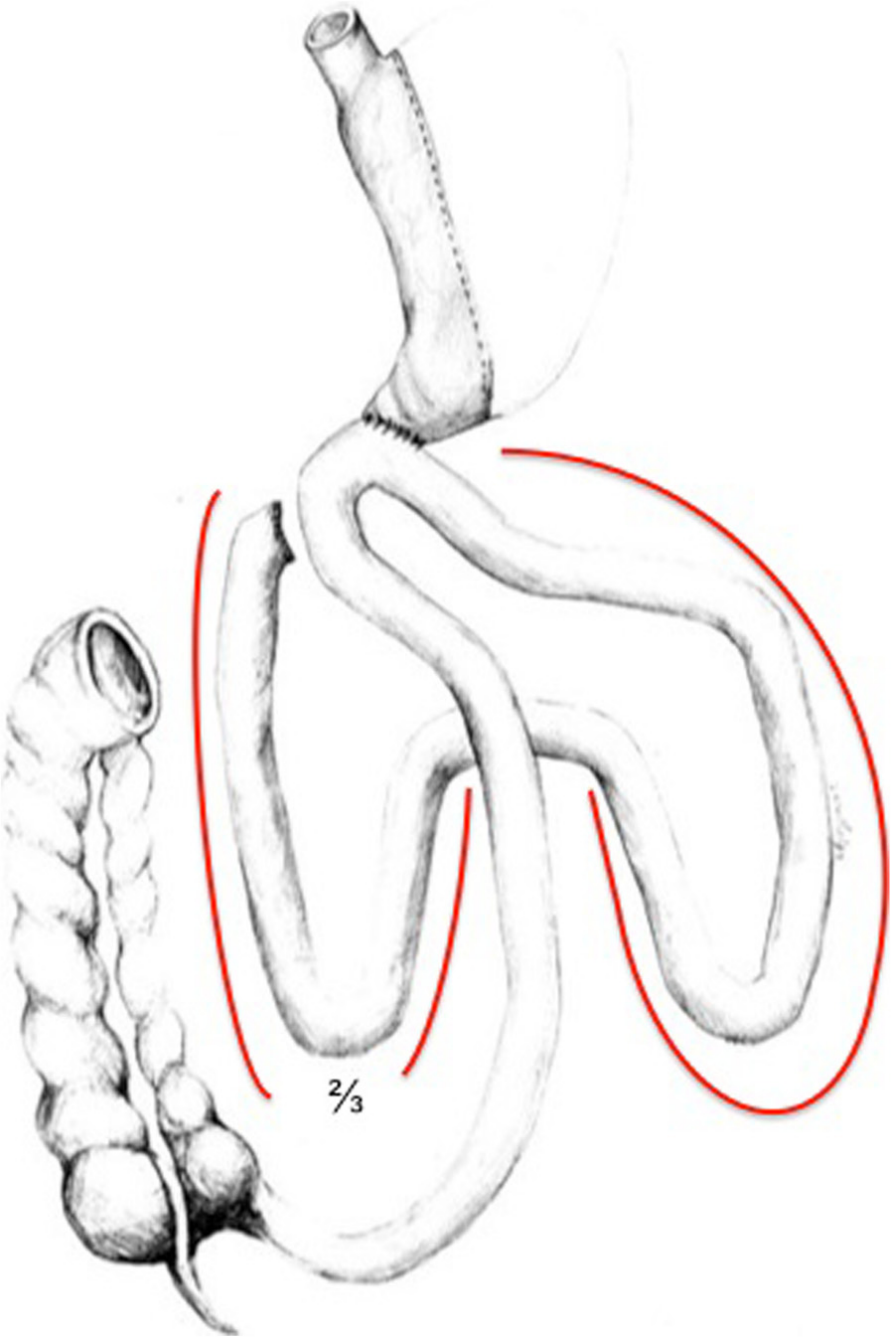
**Diagram of a duodeno-ileostomy with sleeve gastrectomy (DIOS).** The bypassed intestinal length (2/3 of overall intestinal length) is labelled in red.

**Figure 3:**
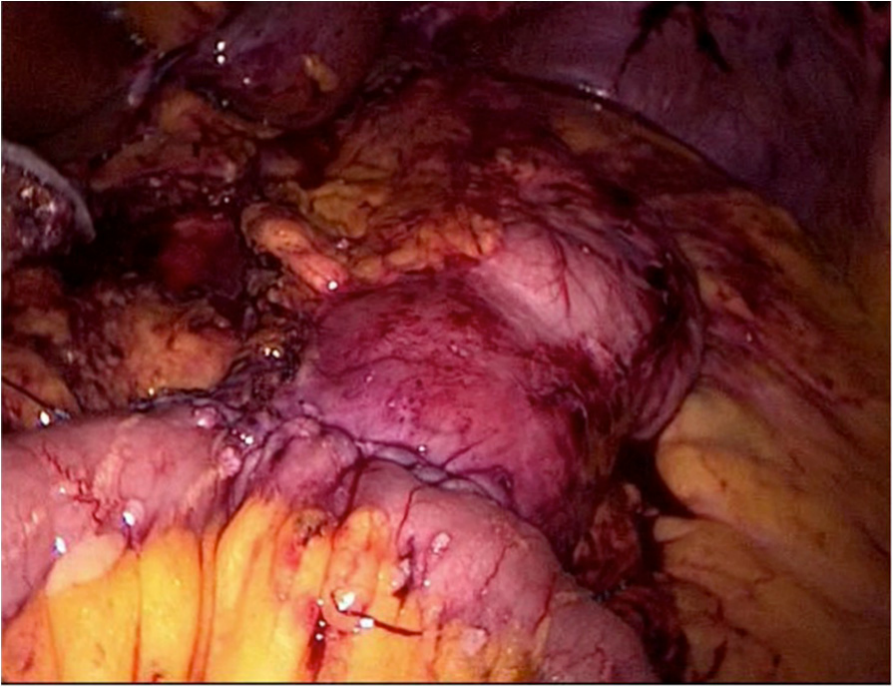
Final aspect of the duodeno-enterostomy.

Sleeve gastrectomy is conducted as described earlier [8]. In case of a stomach plication, we use a modified technique described by Talebpour et al. applying at least two rows of plication using a 3-0 V-Loc™ Suture (Covidien, Dublin, Ireland) [9]. The second part of the operation (second step, when performing a two-step procedure) begins with separation of the duodenum with an endostapling device (GIA- Roticulators, Covidien, Dublin, Ireland, violet cartridge) under preservation of the right gastric artery. Before performing the duodeno-enterostomy, the length of the small bowel is determined to account for inter-individual differences. After measurement, the omega loop should be placed near the postpyloric duodenum with special attention to intestinal alignment to avoid mesenteric malrotation. The position of the duodeno-enterostomy is determined to be aboral to the Treitz ligament, 1/3 of total small bowel length for DJOS (Figure 1), and 2/3 for DIOS (Figure 2). The duodeno-enterostomy is performed as an antecolic, continuous end-to-side hand-sewn anastomosis using 3-0 V-loc™ sutures (Covidien, Dublin, Ireland, Figure 3). Diluted half-strength methylene blue dye (150-200 ml) is used for leak testing. Finally, a drain is put towards the duodenal stump. In case of a two-step procedure, the second part of the operation is conducted separately, then sparing the top left 5 mm trocar needed for sleeve gastrectomy.

### Statistical analysis

Prism 5 for Mac OS X (GraphPad Software, Inc.) was used for all statistical analyses. Statistical significance was set at an alpha of 0.05 for all analyses.

## Results

16 patients underwent laparoscopic DIOS and DJOS operations with a mean duration of 121 min and 147 min, respectively. Overall, 9 patients had undergone previous weight loss surgery, mainly gastric banding (Table 1). A complex gastric reconstruction from RYGB to a sleeve stomach due to uncontrolled dumping syndrome had been conducted earlier in 2 patients. The mean latency between the sleeve and the second step DIOS operation was 17.9 months (Table 1).

**Table 1:** Patient characteristics

	**DIOS**	**DJOS**	**Overall**
Patients	9	7 (gastric plicature: n = 3)	16
Male/Female	3/6	1/6	4/12
Age	52	44	49
Body weight prior to duodeno-enterostomy	114.2 kg (85 – 145 kg)	117.4 kg (103 – 145 kg)	
BMI prior to duodeno-enterostomy	40.63 kg/m^2^ (33.20 – 55.94 kg/m^2^)	41.60 kg/m^2^ (35.74 – 47.90 kg/m^2^)	
Body weight prior to LSG	140.1 kg (105 – 175 kg)		
Gap between LSG and DIOS	17.9 months (3.65 – 41.96 months)		
%EWL before 2^nd^ step surgery	31.73% (-2.67 – 69.54)		
Previous bariatric surgery	5 of 9 (gastric ballon 2, LGB 1, RYGB 2)	4 of 7 (LGB 4)	9 of 16

Values are expressed as means; laparoscopic gastric banding (LGB), Roux-en-Y gastric bypass (RYGB), % excess weight loss (%EWL).

One intestinal perforation occurred upon insertion of the first trocar in a patient with previous gastric banding and subsequent adhesions to the abdominal wall. No complications specific to the duodeno-enterostomy were noted (Table 2).

**Table 2:** Perioperative data

	**DIOS**	**DJOS**	**Overall**	
Surgical complication	0 of 9	1 of 7 (trocar perforation)	1 of 16 (6.3%)	
Operative revision	0 of 9	1 of 7	1 of 16 (6.3%)	
Expansion on surgery	5 of 9 (cholecystectomy 3, appendectomy 2, umbilical hernia repair 1, end-to-end jejunojejunostomy after RYGB 1)	1 of 7 (gastric band removal 1)		
Duration of surgery	120.6 min	147 min		P = 0.112^Ψ^
Length of small intestine	808.3 cm (range: 760 – 850 cm)	701.4 cm (range: 600 – 900 cm)	750.8 cm	P = 0.038^Ψ^
Length of bilio-pancreatic channel	538.3 cm	235.7 cm		
Length of common channel	245.6 cm	465.7 cm		

Values are expressed as means or absolute numbers.

^Ψ^Mann-Whitney test DIOS vs. DJOS.

### Intestinal length

The overall total intestinal length was 750.8 cm (Table 2). Although there was no correlation of total intestinal length and preoperative bodyweight (linear regression p = 0.76), the total small intestinal length in DIOS patients was significantly longer than in DJOS patients (Mann-Whitney P = 0.038, Table 2).

### Weight loss

**Figure 4:**
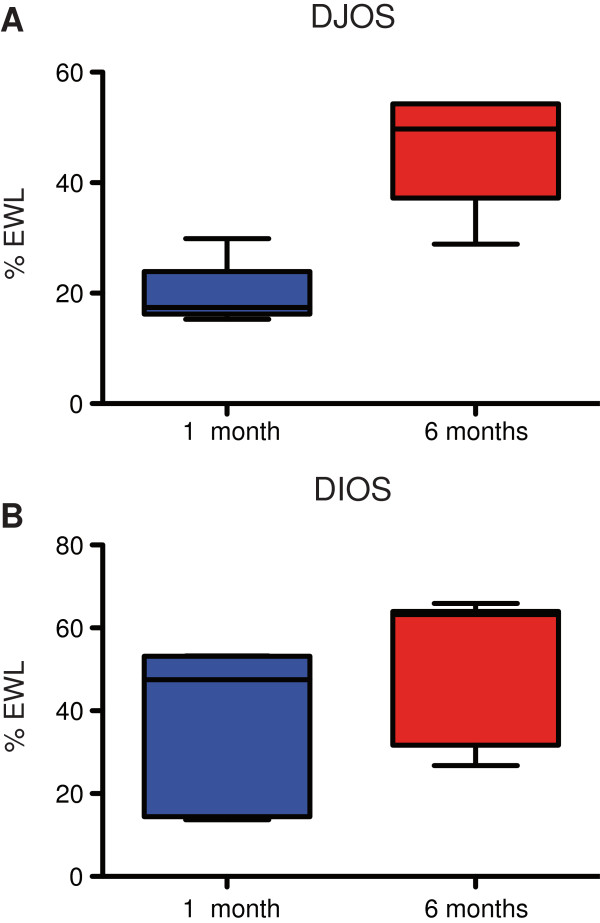
**Box-Whisker-plot of % excess weight loss (%EWL). A**: %EWL of the DJOS and **B**: cumulative %EWL of the two-step DIOS procedure.

The mean preoperative BMI was 40.63 kg/m^2^ in DIOS and 41.60 kg/m^2^ in DJOS patients (Table 1). Patients after primary DJOS operation presented with an excess weight loss (%EWL) of 19.75% and 46.53% at 1 and 6 months (Figure 4A). The overall %EWL of the combined DIOS procedure was 38.31% and 49.60% (Figure 4B). Mean weight loss through LSG alone was 31.73% (range -2.67 - 69.54). Further %EWL came to 18.73% at 1 and 33.03% at 6 months following the second step operation. In this early follow-up, 1 patient did not lose any additional weight after the second step operation despite bypassing 520 cm of small intestine and clinical signs of malabsorption. Furthermore, control CT sleeve volumetry revealed a small volume of 142 ml at 10 months postoperatively indicating sustained restriction.

### Comorbidities

Prior to LSG, 88.9% of patients suffered from T2DM. At the time of the second-step DIOS operation, 44.4% had remained on anti-diabetic medication, 33.3% on insulin therapy (Table 3). 3 months after completion of the second step, only 11.1% (1 patient) still needed anti-diabetic medication. Glycated haemoglobin levels dropped from 6.8% to 5.7% in DIOS and from 8.0% to 6.9% in DJOS patients 6 months after the operation (both NS). LSG alone led to a relief of arterial hypertension in 50% of DIOS patients, with 3 patients remaining on antihypertensive drugs up to the second step operation. Only 1 patient remained on antihypertensive drugs 3 months after the DIOS operation. Interestingly, this is the same patient who also had to continue insulin treatment.

**Table 3:** Morbidity

	**DIOS**	**DJOS**	
Flatulence	55.6% (all regularly)	71.4% (1 regularly, 4 occ.)	
Dumping	0%	14.3% (all occ.)	
Diarrhoea	66.7% (2 regularly, 4 occ.)	28.6% (1 regularly, 1 occ.)	
Reflux	44.4%	85.7%	P = 0.145^τ^
PPI treatment	100%	71.4%	

Values are expressed as fractions with subgroup differentiation in absolute number (peramphases).

^τ^Fisher´s exact test DIOS vs. DJOS.

Reflux was present in the majority of patients (Table 3) with overall 87.5% of patients requiring proton-pump-inhibiting treatment. Corresponding to the shorter common channel, diarrhoea was present in 66.7% of DIOS and in 28.6% of DJOS patients. Overall, 62.5% of patients complaining of diarrhoea reported only occasional episodes. Occasional episodes of dumping were reported by only 1 patient after a DJOS operation.

## Discussion

Major bariatric surgery combines a restrictive gastric component with a rearrangement of the small intestinal passage. Whenever reconnecting the stomach pouch to the intestine, the pylorus can either be preserved (BPD-DS), or excluded, as it is after common RYGB and Mini-Gastric-Bypass (MGB) [3,10]. In order to preserve the pylorus for a bypass-like procedure, we combined a LSG with an end-to-side duodenojeunostomy – DJOS.

Why should the pylorus be preserved? Historically, this debate was initiated after Watson introduced a pylorus-preserving alternative to the classic Whipple procedure in performing a pancreatic head resection [11]. This modification should prevent the patient from typical post-gastrectomy symptoms such as dumping, diarrhoea and dyspepsia [12]. A prospective randomized trial comparing the two procedures could later demonstrate an increased quality of life regarding appetite, nausea and diarrhoea resulting in a faster regain of bodyweight [13]. For bariatric surgery, Hess et al. demonstrated a reduction of marginal ulcers by 90% and no dumping syndrome when the pylorus was preserved during a BPD/DS [14].

Postpyloric anastomosis furthermore allows for loop reconstruction, whereas a “prepyloric” gastro-entero anastomosis necessitates rerouting the biliopancreatic fluids via a foot-point or a Roux-en-Y reconstruction to avoid biliary reflux. Disregarding this principle, surgeons use a loop reconstruction without rerouting biliopancreatic fluids in performing a MGB [10,15]. Although this operation enables excellent weight loss with a low complication rate, operative revision after MGB was mostly due to internal bile reflux and marginal ulcers [16]. Marginal ulcers furthermore also occur after conventional RYGB in about 13% of patients, even though a Roux-en-Y reconstruction had been performed in these patients [17].

Furthermore, pylorus preservation leaves a physiological control mechanism of food output into the small intestine preventing a dumping syndrome [18]. Dumping syndrome is an important issue after RYGB and the overall incidence may rise up to 75.9% [19]. Recently, a detailed examination of postoperative dumping syndrome showed severe problems of fatigue in 12% of patients 2 years after RYGB [20]. The current analysis of postoperative bowel habits after DJOS operations revealed that only 1 patient complained of occasional dumping-related symptoms.

Surprisingly, reflux was present in 86% of patients after the DJOS operation, despite PPI treatment and grossly asymptomatic patients preoperatively. Although we cannot test for this hypothesis, we believe that reflux is a consequence of the LSG in which it is a common phenomenon [21]. However, the reflux incidence in our own isolated LSG collective is much lower and other authors report an incidence of 25-47% [21,22]. We earlier demonstrated a thoracic sleeve migration as a cause of reflux after LSG [23]. Although performing only a limited duodenal mobilisation maintaining the right gastric artery, disruption at the duodenum and pyloric mobilisation may facilitate such a migration. Long-term follow up with close attention on reflux including structured analysis such as 24-hour pH-manometry will further clarify the cause of increased reflux and show, whether these high numbers indeed prove to be an obstacle after DJOS operations.

In the current series, a gastric plicature was used in 3 patients after previous gastric banding. This constellation is a known risk factor for sleeve leak when performing a conventional LSG [24,25]. Gastric plicature has been introduced by Talebpour et al. as an alternative to LSG [9]. Weight loss through this technique alone may be inferior to conventional sleeve gastrectomy and randomized controlled trials have not been conducted to date [26]. However, in case of previous gastric banding and relevant perigastric scar tissue, a gastric plicature may pose an alternative to LSG as the stomach and surrounding scar tissue has not to be cut, especially when combined with additional bariatric options such as a rerouting of the intestine. Certainly to date, this option is relevant only for individual patient cases.

This early follow-up period of 6 months in a small and heterogeneous group does not allow for valid evaluation of weight loss capacity, yet weight loss noted after DJOS is within the range reported by others following RYGB [27,28]. Overall %EWL of DIOS patients was considerably lower when compared to Sachez-Peraut´s SADI-S collective, yet again the majority of patients had undergone pervious weight loss surgery which is known to considerably effect weight loss [7].

Weight regain is a major problem after conventional gastric bypass with up to 35% of patients experiencing an %EWL less than 60%. Causes noted are dilatation of the gastric pouch or enlargement of the gastro-jejunal anastomosis [29]. Clinically, we and others observe that patients regaining weight after RYGB have often lost their feeling of satiety and subsequently consume large meals [5]. We speculate that the DJOS operation has two distinct advantages targeting these drawbacks after conventional RYGB: 1. Anastomotic dilatation will be prevented through pyloric physiological muscle calibration and, 2. LSG is known to create an excellent feeling of satiety due to a deceleration of food transit in the longitudinal part of the sleeve stomach [30].

The major predicament in analysing limb-length variations is the fact that surgeons creating a gastric bypass commonly measure the alimentary and biliopancreatic limb but neglect the common channel length. Surgeons forming a BPD, by contrast, determine the length of the common channel and alimentary limb, and in turn neglect the length of the biliopancreatic limb. Furthermore, the total small intestinal length is highly variable and ranges between 4 to nearly 10 meters [31]. We measured a comparable range of small bowel length of 6-9 m, yet the length variation of bowel measured was large. Compared to the historical measurement of small bowel length in lean adults, the total small bowel length of obese patients was comparable to lean individuals [32].

Establishing the loop duodeno-*jejunostomy* the key issue was to determine an adequate anastomosis position. In conventional RYGB, weight loss is mainly due to calorie restriction, which is substantially caused by appetite control due to a modulation of entero-endocrine peptides, mainly located in the terminal ileum [33]. As this modulation is well known for current limb length variations in RYGB, the focus was to find an anastomosis position similar to conventional RYGB. Here, the alimentary limb ranges from 75 cm to 150 cm with a biliopancreatic limb length of approximately 30 cm [34]. Randomized controlled trials suggest that a long alimentary limb (150 cm) might be preferable for the super-obese [35,36]. However, Stefinidis et al. reviewing the “Importance of the Length of the Limbs for Gastric Bypass Patients” find no clear recommendation [34]. In MGB, the gastro-jejunostomy in usually formed at 150 cm [10]. Some authors suggest an increase in length by 10 cm for every BMI point above 40 kg/m^2^ (MGB for all) [15].

To account for inter-individual differences, as outlined above, we decided to place the duodeno-jejunostomy after 1/3 of small bowel length, bypassing an average of 236 cm in performing a DJOS operation. Taking into account that the loop reconstruction combines the alimentary and biliopancreatic limb, DJOS resembles a long limb RYGB (150 cm plus 30 cm).

For the malabsorptive DIOS operation, the adequate anastomosis position had to be carefully selected in order to prevent excessive malabsorption. As there is no alimentary limb when conducting a loop reconstruction, the common channel had to be considerably longer than in classic BPD surgery. Sanchez-Pernaute et al. extensively reviewed limb length variations when initially describing the SADI-S operation, ultimately deciding to form a common channel of 200 cm [37]. As the SADI-S operation has proven to be safe and effective with no relevant malabsorption in mid-term follow up, anastomosis position for the DIOS operation should be similar [7]. Yet, provided that the overall proportional energy resorption of food consumed does not grossly depend on bowel length, it seems consistent that leaving a 200 cm common channel at a total bowel length of 500 cm causes greater possible malabsorption than the same common channel at 900 cm overall bowel length.

Based on the considerations above, we decided to place the duodeno-ileostomy after 2/3 of the small intestine, leaving a common channel of 1/3. For maximum safety, the common channel was never under 200 cm in length. The 2/3 position left a mean common channel length of 245 cm, thus approximately 20% more compared to the SADI-S operation [7].

## Conclusions

Although two different metabolic principles underlie the DJOS and DIOS operation, performing loop duodeno-enterostomies with sleeve gastrectomy essentially breaks down bariatric surgery into exactly these two distinct elements, leaving the possibility for individual adaptation. The early results of this small and heterogeneous series most importantly show no mortality and no complication related to the duodeno-enterostomy. Pylorus-preserving duodeno-enterostomies with sleeve gastrectomy may open new technical alternatives in bariatric surgery. If the DJOS and DIOS operations prove to be beneficial will have to be evaluated in randomized controlled trials.

## Abbreviations

BMI: Body mass index; BPD: Biliopancreatic diversion; BPD/DS: Biliopancreatic diversion with duodenal switch; CT: Computed tomograpy; DIOS: Duodeno-ileostomy with sleeve gastrectomy; DJOS: Duodeno-jejunostomy with sleeve gastrectomy; LSG: Laparsocopic sleeve gastrectomy; MGB: Mini gastric bypass; RYGB: Roux-en-Y gastric bypass; SADI-S: Single anastomosis duodeno-ileostomy associated to a sleeve gastectomy; %EWL: Excess weight loss.

## Competing interests

Jodok Matthias Grueneberger, Iwona Karcz-Socha, Goran Marjanovic, Simon Kuesters, Krystyna Zwirska-Korczala, Katharina Schmidt and W. Konrad Karcz have no conflicts of interest.

## Authors’ contributions

MG, GM, SK and KK participaed as surgeons for DIOS and DJOS operations, MG, KS and KK have drafted the manuscript, IK-S and KZ-K critically revised the manuscript. All authors read and approved the final manuscript.

## Pre-publication history

The pre-publication history for this paper can be accessed here:

http://www.biomedcentral.com/1471-2482/14/20/prepub
